# Implicit-descriptor ligand-based virtual screening by means of collaborative filtering

**DOI:** 10.1186/s13321-018-0310-y

**Published:** 2018-11-22

**Authors:** Raghuram Srinivas, Pavel V. Klimovich, Eric C. Larson

**Affiliations:** 1 Department of Computer Science and Engineering, Bobby B. Lyle School of Engineering, Southern Methodist University, 3145 Dyer Street, Dallas, TX 75205 USA; 2 DataScience@SMU, Dallas, 75205 TX USA; 3 The Dedman College Interdisciplinary Institute, 3225 Daniel Avenue, Dallas, TX 75205 USA

**Keywords:** Ligand-based virtual screening, Collaborative filtering, Drug discovery, Computational pharmacology

## Abstract

Current ligand-based machine learning methods in virtual screening rely heavily on molecular fingerprinting for preprocessing, i.e., explicit description of ligands’ structural and physicochemical properties in a vectorized form. Of particular importance to current methods are the extent to which molecular fingerprints describe a particular ligand and what metric sufficiently captures similarity among ligands. In this work, we propose and evaluate methods that do not require explicit feature vectorization through fingerprinting, but, instead, provide implicit descriptors based only on other known assays. Our methods are based upon well known collaborative filtering algorithms used in recommendation systems. Our implicit descriptor method does not require any fingerprint similarity search, which makes the method free of the bias arising from the empirical nature of the fingerprint models. We show that implicit methods significantly outperform traditional machine learning methods, and the main strengths of implicit methods are their resilience to target-ligand sparsity and high potential for spotting promiscuous ligands.

## Introduction

Virtual screening is an automated computational method of filtering candidate ligands based upon their inferred relationship with a given target. Screening virtually has a number of cost saving advantages over high throughput screening methods and is a vital part of the drug discovery process [1–5].

However, the cost saving advantages of virtual screening must be reconciled with their ability to accurately find ligands with desired properties from relatively few examples. This is especially true for methods that employ traditional machine learning algorithms to predict binding affinity. The ability of traditional machine learning algorithms to effectively predict binding affinities against a specific target depends on the number of ligands assayed for that target. This is problematic for traditional machine learning algorithms because they generally require many training examples before they can predict the outcome reliably. Moreover, from these relatively few examples, the virtual screening algorithm must score candidates well early in the process of ranking (i.e., the early recognition problem).

To our knowledge, all existing works employ the use of explicit structure featurization in order to “fit” the problem into the workflow of traditional machine learning modeling. These works rely heavily on the cheminformatics tools, such as RDKit [6], to featurize ligands into a finite number of descriptors that characterize the ligandś geometry and physicochemical properties. Explicit prior knowledge of the ligand’s physical structure (either two- or three-dimensional) and its chemical formula is a necessary condition for featurization [7–9].

While knowing the ligand structure is not typically a limiting problem (at least for the case of two-dimensional representation), the reliance on traditional machine learning algorithms to map from these explicit features to a desired outcome requires many training examples. More reliable mapping comes at the expense of needing more training assays so that the machine learning model can learn the relevant portions of the features for the desired task.

In this work, we mitigate the need for large datasets of dense assay examples by adopting an “implicit” structure model of the ligands. That is, for a given ligand, we use the assay results of other implicitly similar ligands to help predict if a particular ligand binds to a target. The measure of similarity is only based on the results of the recorded assays, not featurized descriptors of the ligand. By using this implicit similarity, we can more readily predict if a ligand will bind to a target with far fewer training examples per target. In this way, our method can better understand the implicit structure of a given ligand using assays from another ligand. Thanks to the availability of well-curated and constantly growing databases of assay outcomes such as ChEMBL [10], implicit structure methods can increase their ability to effectively predict binding affinity to targets with sparse assay examples from the sheer volume of other assay examples.

To model implicit structure through similarity, we choose to explore a machine learning method known as collaborative filtering. Collaborative filtering [11, 12] is a technique widely used to develop recommender systems, the algorithms designed to predict the interests of a user based on the analysis of the preferences from many users. For example, when deciding to recommend a movie to a particular user, collaborative filtering is a means for selecting other similar users, then using the ratings of these similar users to predict if the user might enjoy a particular movie. The implicit “preferences” of the user are modeled with collaborative filtering. In this work, we investigate various methods of collaborative filtering for their utility in inferring binding affinity for virtual screening. In particular, our contributions include:

1. An investigation into how collaborative filtering methods can be used to predict binding affinity of ligand-target pairs. We compare collaborative filtering methods to traditional machine learning methods that use explicit fingerprinting from the RDKit package, showing collaborative filtering performs on-par with other methods in terms of all evaluation criteria, including enrichment factor even without the knowledge of explicit ligand’s explicit physical structure.
2. An evaluation of collaborative filtering categorized by the amount of required training assays needed for a target of interest, showing that collaborative filtering has a significant performance advantage when the number of training assays for a given target is relatively low.
3. An introduction of “Implicit Target and Ligand Fingerprints”, a new type of ligand fingerprinting derived from the latent factors employed by the collaborative filtering method.

## Methods

Many previous research approaches have investigated methods for virtual screening. We categorize related work in this section through its usage of traditional machine learning methods. Methods that employ complex physical simulation, such as docking, are grouped as “Alternative Methods” as they share the least amount of overlap in methodology to our work. We discuss machine learning methods in more detail to help distinguish and motivate our work.

### Alternative virtual screening methods

Virtual screening methods that employ detailed physical simulation of the binding of ligands and targets are typically called “structure-based methods,” docking being the most prominent exemplar of such a method. Docking methods consist of physically modeling the binding site of a target and scoring how well a ligand binds to the site in various poses [13]. The performance of a docking tool depends overwhelmingly on the scoring function used and methods for assessing the binding site and ligand structure. Until recently, these methods were limited because the scoring function was predetermined by the developers of the docking software, which made it difficult or impossible to improve the performance by enlarging the training dataset [13–15]. However, the advances of machine learning in drug discovery made it possible to develop ML-based scoring functions which noticeable outperform classical, or expert-based, scoring functions [16].

### Machine learning for virtual screening

*Molecular fingerprinting* Molecular fingerprints have become the basis of ligand-based virtual screening whose requirement is that the molecular structural and physicochemical features are to be represented in the format comprehensible by a computer program, such as a binary vector of predetermined length. Fingerprint types are classified into several families based on the underlying feature-to-numeral mapping algorithm:

- Dictionary-based fingerprints, often referred to as keys. In this method, the structure of the molecule to be fingerprinted is inspected for the presence (or absence) of certain structural fragments from the predefined list, the resulting fingerprint vector elements being ones (if the molecule contains the structural fragment) and zeros (for the substructures not found in the molecule). Examples of the fingerprints belonging to this family are MACCS keys [17] and PubChem keys [18], comprised of 166 and 883 keys, respectively.
- Topological fingerprints. These include linear [19], atom pair-based [20], dendritic [19], and torsional fingerprints [21]. These fingerprints encode the types of molecule’s atoms and paths between them.
- Circular or radial fingerprints [22]. The structural features to be encoded by these fingerprints are constructed by iterating through all atoms and picking an atom and including its atomic surroundings within *N* bonds. Whether the focus is on the atom types (extended-connectivity fingerprints) or pharmacophoric features (functional-connectivity fingerprints), the resulting fingerprints can be represented as bit strings and count vectors.

As of today, extended-connectivity fingerprints with the radius 2 (and the diameter of 4) represented in a form of a bit string (ECFP4 [22]) is one of the most popular fingerprints in virtual screening. Its popularity is owed by several benchmark studies [23, 24] whereby it was shown to outperform other fingerprinting methods. It is this fingerprinting method that we used as the explicit-descriptor baseline when comparing with our implicit-descriptor method based on collaborative filtering.

*Models using explicit fingerprinting* A common method used to evaluate explicit fingerprints is to perform a similarity search of a particular ligand which is then linked to the ligand activity towards a given target. A number of similarity distance metrics, such as Tanimoto [25–27], are used for such quantification. The proliferation of widespread, publicly available cheminformatics tools and molecular fingerprint similarity search algorithms [6, 28] dramatically boosted the number of publications in the ligand-based virtual screening domain [29]. Many researchers have investigated the use of a number of different machine learning models with explicit fingerprints. In most cases, this modeling consists of building a machine learning model for each “target” in the dataset. Ligands that have been assayed with this target are used as training data and the label of the binding affinity is typically made to be binary. That is, two thresholds are applied to the reported binding affinity value in order to separate the ligands into “actives” and “non-actives.” Binding affinities that are in the range within the “actives” and “non-actives” threshold are typically discarded. Using these assumptions, the reported machine learning techniques include random forests [30–34], support vector machines [8, 35–37], k-nearest neighbors [38], naïve Bayes [39, 40], extreme gradient boosting [41], influence relevance voting [42], and shallow artificial neural networks that are typically two or three layers in depth [43–45].

Some works, rather than convert to problem to binary, opt to model the binding affinity value as a multivariate regression problem [46]. Even so, these works often compare the regression output to a static threshold to evaluate the accuracy of the methods resulting in a binary decision problem. There are also a number of works that do not rely on training a model for each specific target. For instance, Ramsundar et al. and Dahl et al. used multi-task neural networks [47, 48]. In these works, the initial layers of the neural network are shared and trained using all the target-ligand pairs in the dataset. The outputs of these shared layers are then fed into other neural network layers that are trained separately for each target. These methods can also be applied to traditional machine learning models, not just neural networks [49]. These methods are typically more resilient to targets with fewer training examples because the shared layers are trained with many examples. Even so, many training examples are required to refine the non-shared parameters for each target. Multi-task methodology, while having some overlap with our approach in that the analysis is performed on multiple targets simultaneously, is nevertheless different as it relies on the ligand and target fingerprint.

Finally, recent work has investigated methods that employ vectorization of the targets and compounds [50]. In this approach, one machine learning model can be trained using the fingerprints for both the target and compound as input vectors. Theoretically this could reduce the training overhead for targets with fewer assays because the global machine learning model learns how to generalize its knowledge of other targets based on their fingerprint. However, this advantage has yet to be established in the literature. Moreover, these methods are fundamentally limited by the quality of the fingerprinting of the ligands and targets. Current methods employ proteochemometric analysis [51], but other methods for fingerprinting have yet to be analyzed in conjunction with machine learning models. The work by Petrone et al investigates the application of using the biological activity data from past assays for comparing compounds and there by developing a set of biological descriptors, termed “high-throughput screening fingerprint” (HTS-FP) [52]. The Z-scores of the percent inhibition values are calculated to derive a vector of a compound’s normalized inhibition values across all assays to create a compounds HTS FP. While their methods also do not rely on the explicit molecular structures, they differ from our work in terms of the core algorithms used and the results achieved as described in subsequent sections. Additionally, the superior performance demonstrated by the collaborative filtering algorithm discussed in this paper with its results for the targets with limited assays (< 100) further differentiates our work from Petrone et al.

We also note that our methods share some overlap with the methods of kernel-based collaborative filtering [53] and multi-task learning [47] that have been applied to binding affinity prediction. The collaborative filtering algorithm in the study by Erhan et al. [53] is based on the JRank kernel perceptron algorithm. The basic idea of the algorithm is to combine collaborative filtering and molecular fingerprinting through a kernel function and a multi-layer perceptron neural network. This has the effect of unifying the target and compound features in a joint feature space in which distances (inner products) can be computed easily. Erhan et al. also investigated using only kernel-based collaborative filtering, but their best results were attained with an explicit featurization process (fingerprinting). Moreover, the reported results of Erhan et al. were not conclusive, with evaluation scores that did not always support the use of collaborative filtering. We hypothesize this was due to the amount of data available in their study (only 24 targets were available for investigation), which limits the expressiveness of collaborative filtering. In our study, we see a distinct advantage in collaborative filtering (albeit we use different filtering methods and have a larger dataset).

### Overview of collaborative filtering

Most virtual screening mechanisms have relied on the structural information of ligands and/or targets. We propose an extension to these techniques by incorporating the concept of collaborative filtering. Collaborative filtering algorithms have been historically used in the context of designing recommendation systems such as movie recommendation engines, as well as up-sell and cross-sell recommendation engines for e-commerce sites. In general, collaborative filtering is a method for making automatic predictions (filtering) about the interests of a user by collecting preferences or taste information from other users (collaborating) [54]. This approach relies on modeling predictions using past interactions between the users and the items rated. This is in contrast to traditional machine learning that models individual users or items based on their attributes. For example in the context of the movie recommendation application, a movie could be described by its genre, reviews, starring actors, and awards, and a user could be described by her/his demographic information, any past reviews, genre preferences, friends reviews, and so on. It quickly becomes evident that identifying the entire gamut of properties that accurately represent the users and movies is an intractable task. Collaborative filtering, then, is an alternate technique which relies on past transactions without relying on explicit attributes of the user or movie. We extend this concept to the domain of virtual screening where we liken the targets to users and ligands to items. The “rating” between targets and ligands can be represented by the known binding affinities (active or inactive). The assays and their results reported in the ChEMBL database serve as a useful source of interaction data for collaborative filtering.

In general, collaborative filtering methods can be categorized into two groups of methods: the neighborhood methods [55] and matrix factorization methods [56], also known as latent factor models. Neighborhood methods compute the relationship between items and/or users to identify similar items or like minded users to help predict ratings. Latent factor methods try to explain the ratings by characterizing the items and users on 20–100 factors, derived entirely from past rating patterns.

#### Neighborhood-based collaborative filtering

Neighborhood methods (also called memory-based methods) evaluate the relationships between items and users by approximating the relative distance between users. In this scenario, there is a large user-item matrix, *A*, with users in the rows and items in the columns. A particular rating between a user, *u*, and item, *i*, is denoted as $a_{u,i}$. This matrix is typically sparse, with only a handful of ratings for each item per user. The general concept is to find similar users by taking the similarity among each row of *A*. In this method the system evaluates a user’s preference for an item based on ratings of similar users that have also rated that particular item [55]. More formally, we define this process for a particular user, $u_0$, and item, *i*, as:

$$a_{u_0,i} = \frac{1}{|U|}\sum _{u\in U} a_{u,i}$$

where *U* is the set of all similar users that have also rated item *i* and |*U*| is the total number of similar users. Variants of this measure also exist where the $a_{u,i}$ measure is weighted, for example, by the relative similarity of users. Similarity of users can be calculated using various distances. Common measures of distance include Euclidean, Cosine, and Pearson Dissimilarity. Variants also exist on the “rules” for judging similar users. Some methods look for the top-N similar users, whereas other methods employ a distance threshold for discerning which users are similar. In general, neighborhood methods tend to work well for a number of different applications, but suffer from computational issues when the user item matrix *A* is large or very dense.

#### Matrix factorization method

**Fig. 1:**
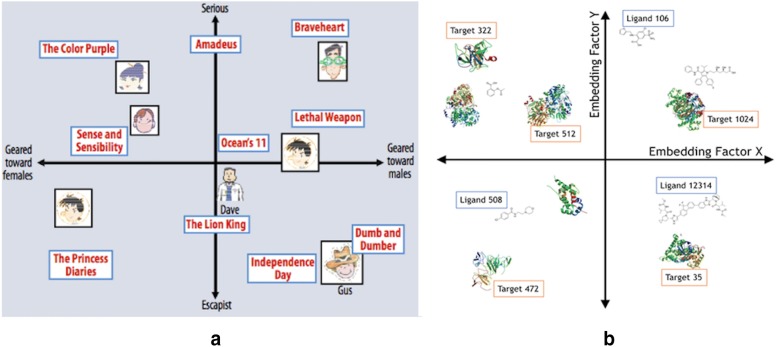
Latent factor embedding. Sub figure a illustrates the concept of latent factor the latent factor recommendation for a movie recommendation engine. The latent factor method relies on learning hidden factors from the user-movie ratings alone. In this simplified example the system learns two dimensions from the ratings and places the movies and users in this 2D space. The users predicted rating of the movie would be a dot product of the user’s and movie’s location in the 2D space. Figure b illustrates the same concept for a target-ligand embedding. Here the latent factors correspond to properties that the ligands and protein targets can be jointly modeled with. The properties may correspond to a distinct chemical property, but might also pertain to a factor not well described by traditional cheminformatics

Matrix factorization methods [54] have been one of the most popular implementation techniques of latent factor recommendation systems. These methods find lower dimensional representations of the full user-item matrix. The dimensions of the lower dimensional representations are often called factors. In the context of movie recommendation engines, the discovered factors from matrix factorization methods have been studied extensively. While there is no guarantee that the factors found represent an interpretable quantity, many times the factors can be identified as representing a number of interesting item and user properties (even though the modeling does not explicitly use any features of the user or item). For example, in movie recommendations these factors often “encode” obvious factors such as comedy versus drama, amount of action, or orientation to children. They can also represent less well-defined dimensions such as depth of character development, quirkiness, or they might be completely uninterpretable dimensions. For users, each factor represents how much the user likes movies that score high on the corresponding movie factor [54]. For the target-ligand application, then the factors might encode properties of the binding sites for the target or chemical properties of the ligands. We illustrate the metaphor in Fig. 1.

When applied to the domain of virtual screening, this method involves representing the ligands and targets as vectors of factors with dimensionality *f*, to represent the latent factor space, where $A\approx {\hat{A}}=P\cdot Q$. Here the affinity predictions are modeled using the well established singular value decomposition method [57] and optimization procedures to minimize the reconstruction error between *A* and ${\hat{A}}$. That is, we model each known affinity $a_{t,l} \in A$ between ligand *l* and target *t* as the following dot product of vectors $p_t$ and $q_l$

$$a_{t,l} = p_{t} \cdot q_{l}$$

where each known ligand *l* is associated with vector $q_{l}$ and each target associated with vector $p_{t}$. Both $q_{l}$ and $p_{t}$ contain *f* elements. This operation is often represented in matrix form when there are *L* unique ligands and *T* unique targets in the database:

$${\hat{A}} = P\cdot Q = \underbrace{ \begin{bmatrix} \leftarrow&p_1&\rightarrow \\ \leftarrow&p_2&\rightarrow \\&\vdots&\\ \leftarrow&p_t&\rightarrow \\&\vdots&\\ \leftarrow&p_T&\rightarrow \end{bmatrix} }_{\text {target-factor matrix}} \cdot \underbrace{ \begin{bmatrix} \uparrow&\uparrow&\uparrow&\uparrow \\ q_1&q_2&\dots&q_l&\dots&q_L \\ \downarrow&\downarrow&\downarrow&\downarrow \end{bmatrix} }_{\text {factor-ligand matrix}}$$

**Fig. 2:**
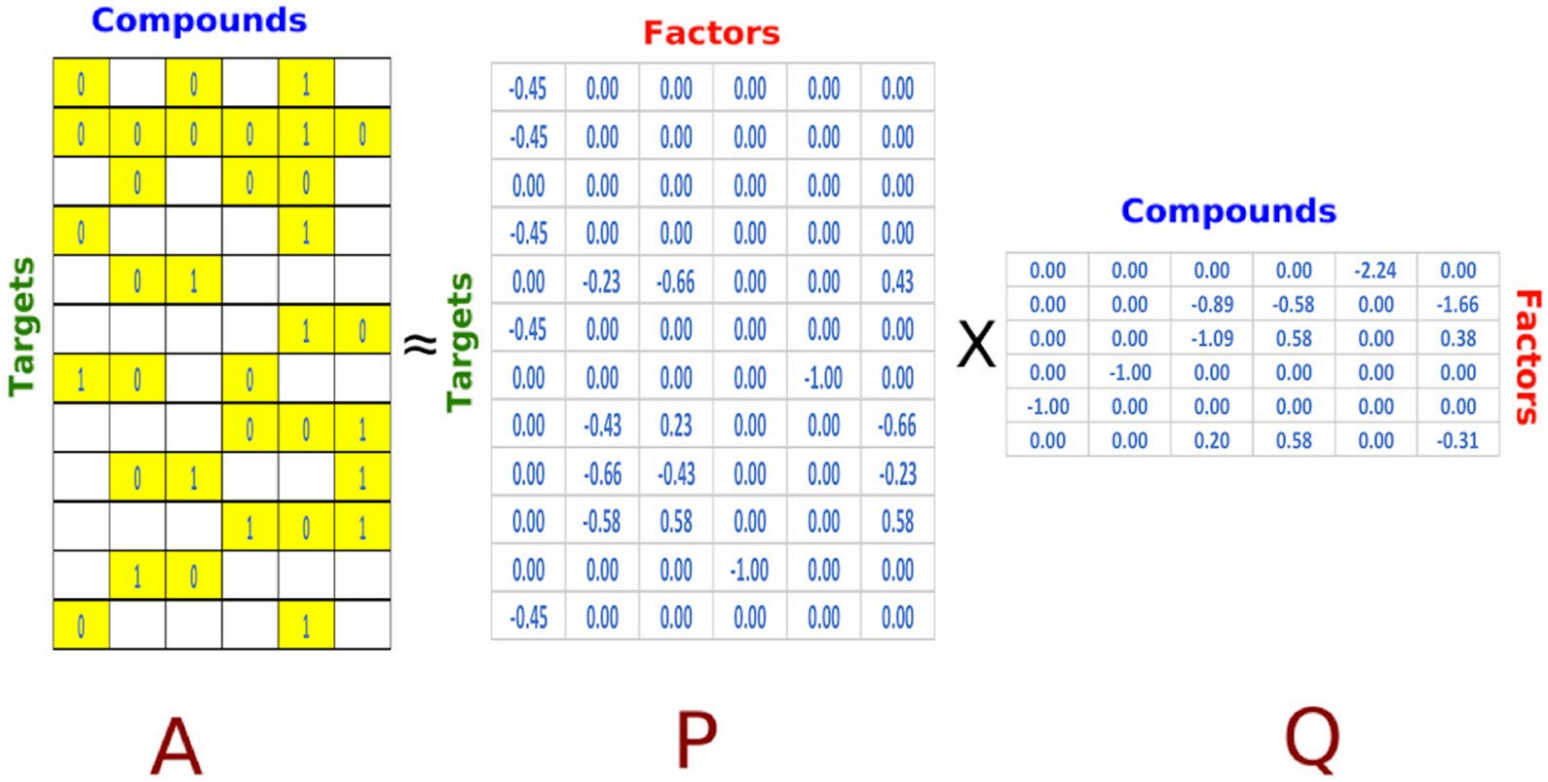
Illustration of the matrix factorization method. In this example the highlighted cells represent the known affinities. The SVD method generates the matrices *P* and *Q*. Optimization methods are employed to minimize the error between the known affinities and their predicted values

The optimization step involves learning the factor vectors $q_{l}$ and $p_{t}$ by minimizing the regularized square error on the set of known affinities, $a_{t,l}$, using standard optimization techniques such as stochastic gradient descent algorithms [58].

$$\min _{p,q} \sum _{t,l\in \Lambda } \underbrace{(a_{t,l} - p_{t} \cdot q_{l})^2}_{\text {mean square error}} + \underbrace{\lambda \cdot (||p_t||^2+||q_l||^2)}_{\text {regularization}}$$

where $\Lambda$ is set of *t*, *l* for which the affinities $a_{t,l}$ is known. The optimization function also includes the regularization term with the regularization parameter $\lambda$ to help minimize over-fitting. Figure 2 illustrates the method with an example. The highlighted cells in the matrix $A_{t,l}$ represents the known affinities between a hypothetical set of 6 ligands and 12 targets. The matrix factorization involves employing single value decomposition [57] method to construct matrices *Q* and *P* with $f=6$ factors in this example. The optimization method involves reducing the square error between the known affinities and the predicted values of the known affinities resulting from $P\cdot Q$. Variants of the factorization methods also exist where the dot product is approximated in a larger dimensional space using kernels, $\kappa (p,q) = \phi (p_{t}) \cdot \phi (q_{l})$, where $\phi$ is a transformation of the vectors into a higher dimensional space. However, it has been previously shown that this is typically poor performing for target-ligand binding affinity prediction [53].

We note that the assay training data in the context of binding affinities can be sparse (as in our case where the observed values of affinities account to .15% of the matrix ). The sparsity in the matrix is due to the unobserved or unknown binding affinities which the algorithm aims to accurately predict.The optimization algorithm learns the factor vectors by minimizing the reconstruction error on the known set of affinities from the training data using the stochastic gradient descent algorithm. Each observed pair of known affinities between targets and ligands from the training set are used to train the model across multiple training iterations. Additionally an adaptive sampling strategy of choosing unobserved pairs complementing the observed affinities is also employed. The algorithm selects candidate negative ligands for the target for which affinities are not known. The algorithm scores each one using the current model and then incorporates the ligand with the largest predicted score for the subsequent training iteration. This adaptive sampling strategy provides faster convergence [59].

### Preprocessing and evaluation methodology

#### Data selection from ChEMBL23

We use ligand-target bioactivity data from the ChEMBL database (Version 23). In an effort to keep the evaluations consistent with previous studies [9],we focus exclusively on human targets and three types of binding affinity measures: half maximal inhibitory concentration ($IC_{50}$), half maximal effective concentration ($EC_{50}$), and the inhibitory constant ($K_i$). When more than one binding concentration measure was present in the database, we use the $K_i$ measure. When $K_i$ is not present we look at $IC_{50}$ and then $EC_{50}$ to categorize the ligand-target pair as active or inactive. To convert data into the binary active-inactive format the following concentration thresholds were applied: $<100$ nM for “actives” and $>1000$ nM as “inactives.” We note that many other works apply two thresholds to the dataset and the interactions between the two ranges are discarded, as their classification is subjective [9, 60]. We also note that the thresholds selected in our study are consistent with the standardized activity values of the CHEMBL database.

The data selection methods described above resulted in a bioactivity matrix of size 241,260 (ligands) by 2739 (targets), with about 0.15% of the matrix containing a real value. The mean inactive:active ligand ratio across targets in the dataset was approximately 7:3.

**Fig. 3:**
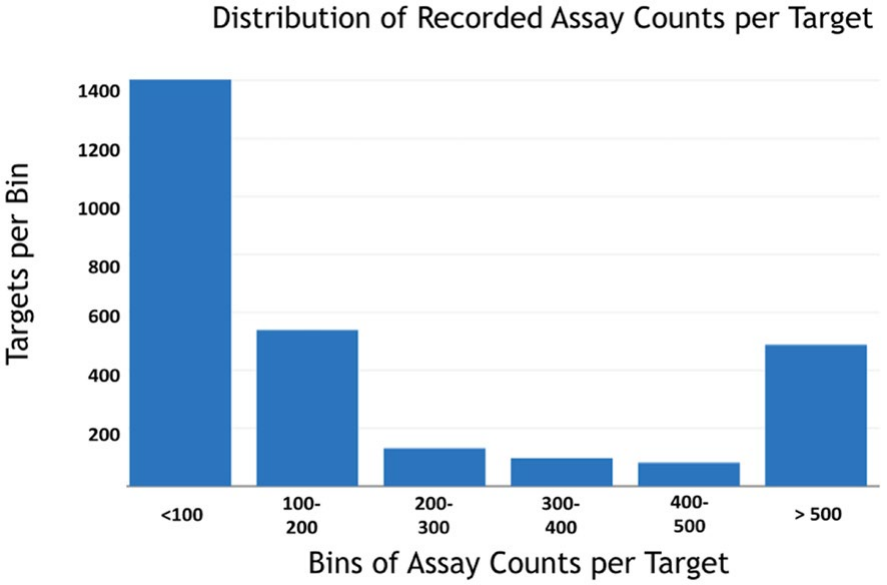
Number of targets by known assay counts. Illustration of the distribution of the number of targets by the known assay counts for each target. More than half of the targets have less than about 200 recorded assays. Therefore, it is imperative that the virtual screening methods perform well when the number of assays is relatively sparse

Figure 3 illustrates the distribution of the targets by the number of recorded assays available for predictions. It was observed that more than half of the targets in the data set had less than 200 recorded assays (including actives and inactives).

We note that there have been recent reports in the literature [60, 61] stressing that the overall model performance is highly dependent on threshold selection for inactive/active as well as the ratio of inactive to active examples. Also, many ligand-target databases are biased in that the experimental data they are comprised of represents only a small, nonuniform portion of the chemical space. This leads to the over representation of certain types of ligand-target patterns. Furthermore, experimental binding affinity measures are often difficult to reproduce [62], which means there is inherent noise in the datasets such that perfect classification of any test set should not be possible (unless models are over-fitted to the problem). Although this has been a common knowledge within the community [63], it nevertheless remains largely un-addressed.

Following recommendations from [61], we computed the bias of our training and test selection to provide an estimate on how trustworthy our evaluation metrics are.

#### Evaluation criteria

In this study three evaluation criteria are employed: the area under the receiver operating characteristic (AUC), the enrichment factor (EF), and the Boltzmann-enhanced discrimination of the receiver operating characteristic (BEDROC). We briefly describe our rationale in selecting these criteria, as well as an overview of the strengths and weaknesses of each criterion.

Our first evaluation criterion is the area under the receiver operating characteristic curve (ROC [64, 65], the curve that results when the true positive rate is plotted as a function of the false positive rate). This measure ranks ligands based on their predicted probability of being active. An AUC value greater than 0.5 suggests that the classifier is better than chance at assigning an active/inactive target-ligand pair. While widely reported in a number of papers that use machine learning for target-ligand classification, the AUC does not capture important aspects of the virtual screening problem. Specifically, the challenge is to rank active ligands for a given target from the entire dataset. A superior classification model would have a high true positive rate for the highest ranked ligands, which are the ones that would be assayed first (the so-called early recognition problem). The AUC does not take into account this early recognition, so it can incorrectly judge a classification model superior if it has an overall high true positive rate, even though the true positives may not occur “early” in the ranking of ligands. Such a model would result in many needless assays before becoming sufficiently accurate. Therefore, more suitable metrics are often sought after that do take into account early recognition – the two popular choices being Enrichment Factor (EF) and Boltzmann-Enhanced Discrimination of Receiver Operating Characteristic (BEDROC).

Enrichment Factor [66, 67] is defined as the ratio of correctly classified active ligands within a predefined early recognition threshold compared to the total ligands selected by the model, further normalized by the expected random selection of the ligands.

$$EF_{X\%} = \frac{{\textit{Compounds}}_{{\textit{selected}}}/ N_{X\%}}{{\textit{Compounds}}_{{\textit{total}}}/N_{{\textit{total}}}}$$

where $N_{X\%}$ is the number of ligands in the top $X\%$ of the ranked ligands. $EF_{1\%}$, then, is the ratio of true actives found in the top 1% of ranked ligands from a model normalized by the total number of actives for a specific target. In other words, it gives an estimate on how many more actives can be found within the early recognition threshold compared to a random distribution. While this criterion closely matches the virtual screening problem, it is not appropriate to compare the EF values obtained for different datasets when their number of actives differ. Another disadvantage of the criterion is that it assigns equal weights to the actives within the threshold, without any knowledge that some actives bind extremely well and others have higher $K_i$ concentrations. Robust Initial Enhancement (RIE) [68] helps mitigate this by comparing two scenarios, (1) when the most active ligands are ranked at the beginning of the threshold and (2) when the most active ligands are ranked closer to the end of the threshold. This is achieved by applying continuously decreasing exponential weight when ranking ligands. The RIE metric is similar in meaning to EF in that it quantifies the superiority (to random) of the exponential average of the distribution generated by the ranking method. Its minimum and maximum value dependance on (apart from the pre-exponent factor $\alpha$) the number of actives and the dataset size contributes to the metric disadvantages. Nevertheless, RIE’s desirable property of differentiating actives within the ordered list serves as a driving force for the development of the BEDROC metric, discussed next.

Bound between 0 and 1, the BEDROC metric [69] is interpreted as the probability that an active in the ordered list will be ranked before a ligand that is drawn from a random probability distribution function. The shape of the distribution is governed by the pre-exponent factor $\alpha$, that must be selected by the user. In the words of the original authors: “It is to be noted that $\alpha$ should not be chosen in such a way that it represents the best performance expected by a ranking method, but rather it should be considered as a useful standard to discriminate better or worse performance in a real problem to which the ranking method will be applied.” [69]. Our study chose an $\alpha =20$ based on the previous study by Riniker and Landrum [28] in their benchmarking of fingerprints for ligand-based virtual screening.

**Fig. 4:**
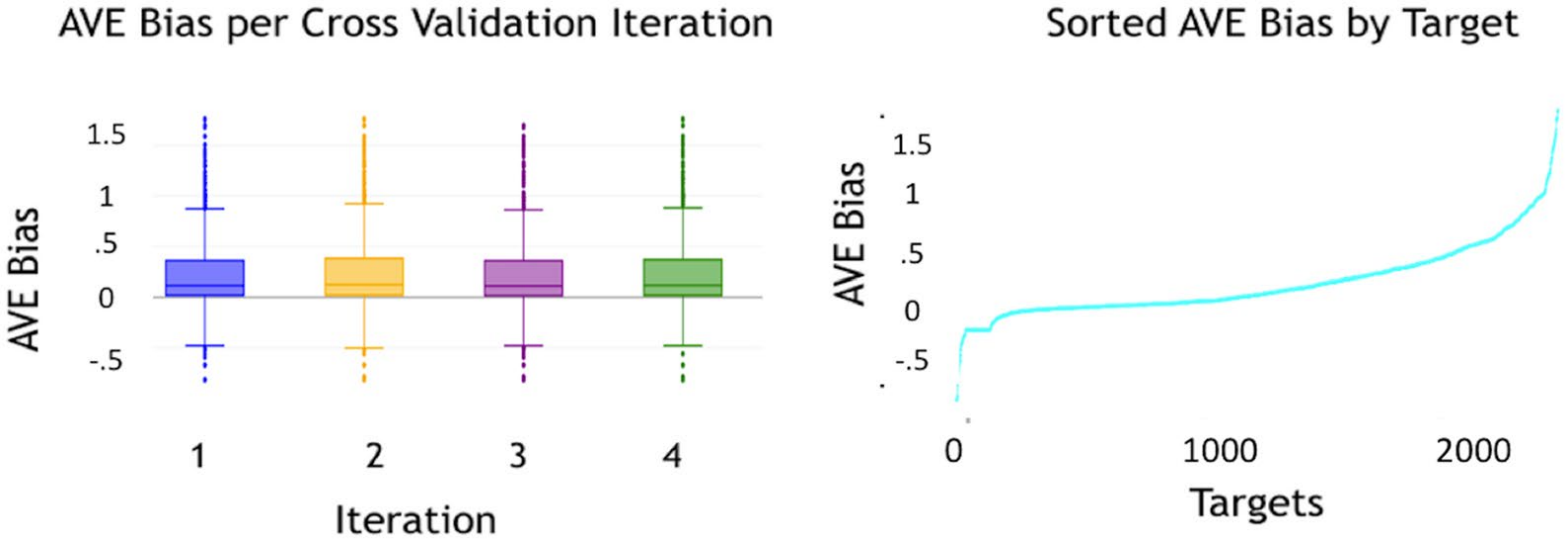
AVE bias. The image to the left visualizes the comparison of AVE Bias across 4 sets of training and validation data. The figure shows that the 4 training and validation sets are randomly split with similar bias measures across the sets, thereby minimizing the impact of variance across our study. The image to the right shows the spread of the AVE Bias for each target in the dataset. The distribution of the AVE Bias from − 0.5 to 1.5 (where closer to 0 indicates no bias) across targets facilitates the study of the resilience of the collaborative filtering algorithm to the impact of such a bias

#### Variance and bias in validation set selection

The performance of machine learning models can be impacted by variance. A model with high variance performs inconsistently on different validation sets. A model with high bias is one that is well fitted to the training data but fails to generalize well. Building machine learning models by using cross validation to separate training and validation can aid in quantifying variance. In our experiments, the train and the test split was randomly generated by stratifying on the targets, generating a random split with the ratio of 70%:30% of associated ligands by target between the training and the validation sets.

In order to minimize the effects of variance influencing the claims in our study, we use four iterations of our tests including generating four sets of training and validation data and building the above mentioned models using implicit and explicit methods for each set of training and validation data. Modeling bias is another design consideration while building machine learning models to ensure the ability of the models to generalize beyond the training and validation datasets. In the field of computational chemistry the accuracy in practice is not as good as the benchmark results from previous virtual screening results [54].

Wallach and Heifets [61] introduced a new measure of evaluating the redundancies in the training—validation sets called the Asymmetric Validation Embedding Bias (AVE Bias). The AVE Bias measures the quality of the training and validation sets by measuring the similarities between the actives and inactives in the validation sets with the actives and inactives in the training sets. The Bias is mathematically defined as [61]:

$$B = \left[ \underbrace{H_{(V_a,T_a)}-H_{(V_a,T_i)}}_{AA-AI}\right] + \left[ \underbrace{H_{(V_i,T_i)} - H_{(V_i,T_a)}}_{II-IA}\right]$$

where, $V_a,V_i$ represent the validation sets with active and inactive ligands, $T_a,T_i$ represent the training sets with active and inactive ligands respectively, and $H_{(\cdot )}$ represents a measure of cluster similarity between the sets. The left $AA-AI$ term of the AVE Bias is a measure of how clumped the validation actives are among the training actives. The right $II-IA$ measures the degree of clumping among the inactives. The study showed that the performance of the ligand based screening methods strongly and positively correlated with the AVE Bias. Intuitively, it means that an algorithm might perform well because there is an inherent difference in the training and validation sets employed that makes the problem more easily separable for the validation set. Positive values indicate a bias, whereas negative values indicate that the problem is increasingly difficult because the training and validation sets differ in a way that makes the classification problem even more difficult. The AVE Bias can be measured for every target in the dataset. Therefore, it is appropriate to present the AVE Bias results as a histogram or boxplot of the values.

As described, we use four stratified shuffle splits of the data. We computed the AVE Bias for all targets in each of the four iterations of train-validation sets. In our calculations, we employed Tanimoto similarity [25] to compute $H_{(\cdot )}$. Figure 4 illustrates the AVE Bias scores using a boxplot to summarize the AVE Bias per target (a separate boxplot is used for each iteration of the shuffle split). All four sets of train and validation sets have similar AVE Bias measures. The imbalanced natured of our data set (inactives to actives, 7:3) in addition to the method of classifying molecules as active or inactive based on the 100 nM and 1000 nM concentration thresholds and the randomized selection of training and validation set resulted in a good distribution of targets with varying AVE Biases across targets. In addition, it was also observed the data set also contained a subset of targets with a negative bias, thus makes the classification of actives and inactives challenging for these sets [61]. Separate plots are shown for each iteration of the validation split. Each box plot represents distribution of the AVE Bias calculated per Target for each iteration of Training and validation sets. Figure 4 also illustrates the AVE Bias for each target in the dataset.

## Results and discussion

In the following section we compare the performance of the implicit-descriptor methods with the performance of explicit-descriptor methods. A number of different variants of collaborative filtering methods were investigated, discussed below. In addition we use two popular classification algorithms that are often employed in virtual screening applications, Random Forests (RF) and K-Nearest-Neighbors (KNN) to comparatively asses the performance of collaborative filtering. For these baseline classification models, we employ two “modes” of training: “per target” mode and a “across targets” mode.

- In the **per target mode**, a machine learning model was trained for each target. The features used for training, then, only consist of fingerprints for the compounds. Because we use four-fold cross validation, this results in four models created for each target.
- In the **across targets mode**, one machine learning model is trained using all targets and ligands. This method requires that we use training features comprised of fingerprints for each compound and fingerprint of each target. Because we use four-fold cross validation, this results in four models trained.

We create explicit structural fingerprinting of the compound using RDKit [6] and we create fingerprints of the targets using ProFeat with principal components analysis. Compound fingerprints from RDKit were generated using 512, 1024, and 2048 bit length sequences. We chose to use fingerprints of length 512 because they performed relatively the same regardless of length (see “Appendix” for this additional analysis). For the target fingerprints, we reduced the dimensionality of the 1447 target features provided by ProFeat to 150 components, which captured nearly 100% of the variation in the data. Additional details on this process are available in the “Appendix”.

All the classification models for explicit structure were implemented using the scikit-learn [70] python library. Hyper parameters of the RF and KNN algorithm were chosen through a randomized grid search, using the Area Under the Curve as the evaluation criteria. The grid search yielded that the Random Forest algorithm was most optimal using 500 total trees. We note that the ensemble tree methods such as Random Forest are typically more robust under data scarcity and our parameter selection is consistent with the previous studies employing random forest classifiers [71]. For the K-Nearest Neighbor algorithm, the grid search yielded that the most optimal parameters were: using brute force distance calculations, “jaccard” distance (which is most appropriate distance calculations in this case as the explicit fingerprints are binary vector), and $k=5$ nearest neighbors. Formally, the upper limit of k is the total number of compounds in the data set; however, the best value from our grid search was found to be in line with previous studies employing this technique [72].

The best performing baseline model was found to be the Random Forest model trained using *across targets mode*, with a median AUC $\approx 0.86$. A more detailed explanation of the baseline results follows later in this section.

**Table 1:** Collaborative Filtering hyper-parameter tuning: Tabulated top 5 results from training multiple collaborative filtering models using neighborhood methods and matrix factorization methods

Distance	N. Threshold	AUC	BEDROC_20_	EF_1%_
Neighborhood-based collaborative filtering				
Pearson	10^−2^	0.791	0.723	4.225
Pearson	10^−5^	0.792	0.723	4.225
Pearson	10^−4^	0.791	0.723	4.225
Jaccard	10^−2^	0.648	0.647	3.670
Cosine	10^−5^	0.644	0.647	3.670

Num. Factors	SGD step size	AUC	BEDROC_20_	EF_1%_
Matrix factorization-based collaborative filtering				
50	10^−3^	0.891	0.929	5.476
32	10^−4^	0.860	0.889	5.028
32	10^−2^	0.899	0.872	4.994
25	10^−4^	0.868	0.867	4.765
25	10^−2^	0.892	0.847	4.547

### Collaborative filtering parameter selection

In order to identify the best performing collaborative filtering model, we employ two randomized grid searches.

The first grid search investigates parameters for neighborhood CF methods. The second grid search investigates parameters for using matrix factorization CF methods. We separate the grid searches because the parameters in each algorithms are quite different. We use collaborative filtering algorithms implemented in the GraphLab API [59].

For the neighborhood CF methods we investigate three separate distance metrics: Jaccard, Cosine, and Pearson. We also investigate a number of threshold values to determine neighborhood size, logarithmically spaced from $10^{-7}$ up to $10^{-1}$. For each combination of hyper parameters, we calculate the mean for each of our evaluation criteria: AUC, $\hbox {BEDROC}_{20}$, and $\hbox {EF}_{1\%}$ scores. The best performing neighborhood CF method utilized the Pearson similarity metric with a threshold of $10^{-2}$. The mean AUC, $\hbox {BEDROC}_{20}$, and $\hbox {EF}_{1\%}$ scores were 0.79, 0.72, and 4.22%, respectively. Table 1 summarizes additional results for the top 5 models, sorted by the $\hbox {EF}_{1\%}$ score.

For the matrix factorization-based CF models, we investigated the number of latent factors, ranging from 5 to 60 in increments of 5. We also investigated the value of the regularization constant, *C*, used in the stochastic gradient descent method [58], with values logarithmically spaced from $10^{-7}$ up to $10^{-1}$. Finally, we swept the values of the step size used for updates in the stochastic gradient descent (SGD) optimization, spaced logarithmically from $10^{-3}$ up to $10^{-1}$. We remind that the factorization CF method learns latent factors for each ligand and for each target and uses them to rank ligands according to the likelihood of observing those (target, ligand) pairs. The stochastic gradient descent algorithm was used as the optimization function to minimize the mean square error between the known affinities and their predictions. Table 1 summarizes the results of the factorization CF method, sorted by $\hbox {EF}_{5\%}$ (SGD step size not shown for brevity). From the table, it is clear that the performance of the factorization CF method exceeds that of the neighborhood recommender. The best model was found with 50 latent factors, a value of $C=10^{-3}$ for regularization, and $10^{-3}$ for the SGD step size. The best model had a mean AUC, $\hbox {BEDROC}_{20}$, and $\hbox {EF}_{1\%}$ as 0.89, 0.92, and 5.47, respectively.

**Fig. 5:**
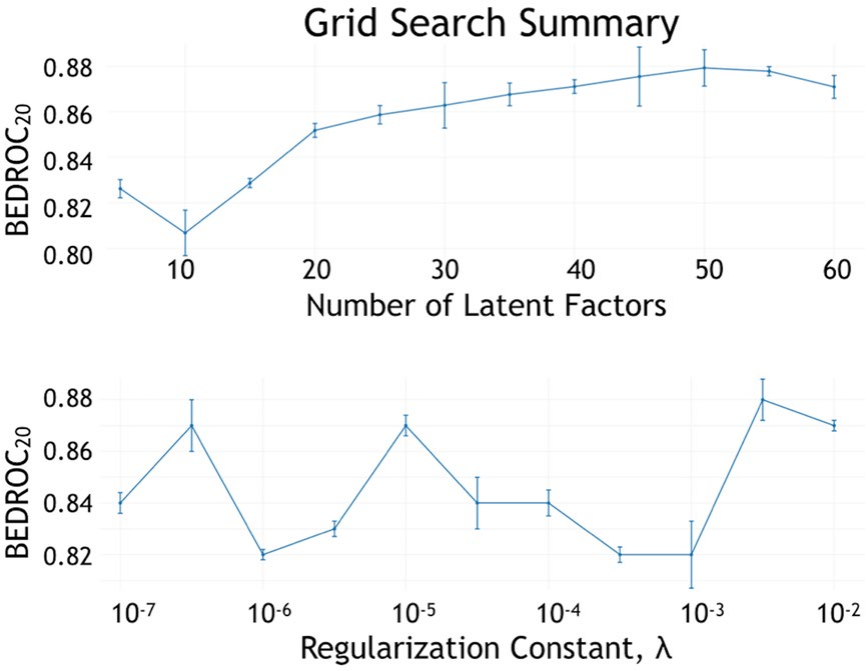
Hyperparameter tuning for matrix factorization collaborative filtering. Illustration of the effects of the number of factors, *f*, and regularization parameter, $\lambda$, on model performance. While model performance improves with more factors, it may lead to over fitting. The regularization parameter does not have a consistent performance, revealing that performance is not sensitive to $\lambda$.The error bars represent the standard error of the mean across the $\hbox {BEDROC}_{20}$ scores for each Target at the specified parameter value

To more clearly understand how the hyper-parameters change the performance of the factorization CF algorithm, we plot the $\hbox {BEDROC}_{20}$ values as each hyper-parameter value changes. Figure 5 illustrates the effect of the number of latent factors on the performance of the model. It was observed that the performance plateaus near 50 factors. Similarly Fig. 5 illustrates the performance of the regularization parameter of the SGD optimizer [58] on the model performance. It is noticed that there is no consistent effect of the value of regularization on the performance of the model.

**Fig. 6:**
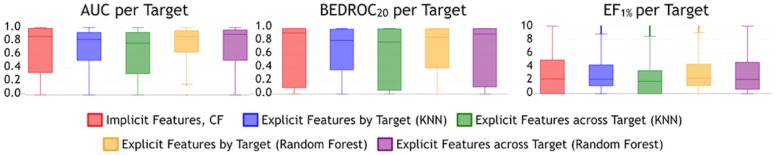
Comparison of AUC, BEDROC_20_, and EF_1%_ scores across all algorithms. The collaborative filtering based implicit structure method, based only on assay outcomes performs on par with the other methods across the three evaluation criteria. The across target Random Forest model is the next best performer. All other models perform about the same and poorer than collaborative filtering

**Fig. 7:**
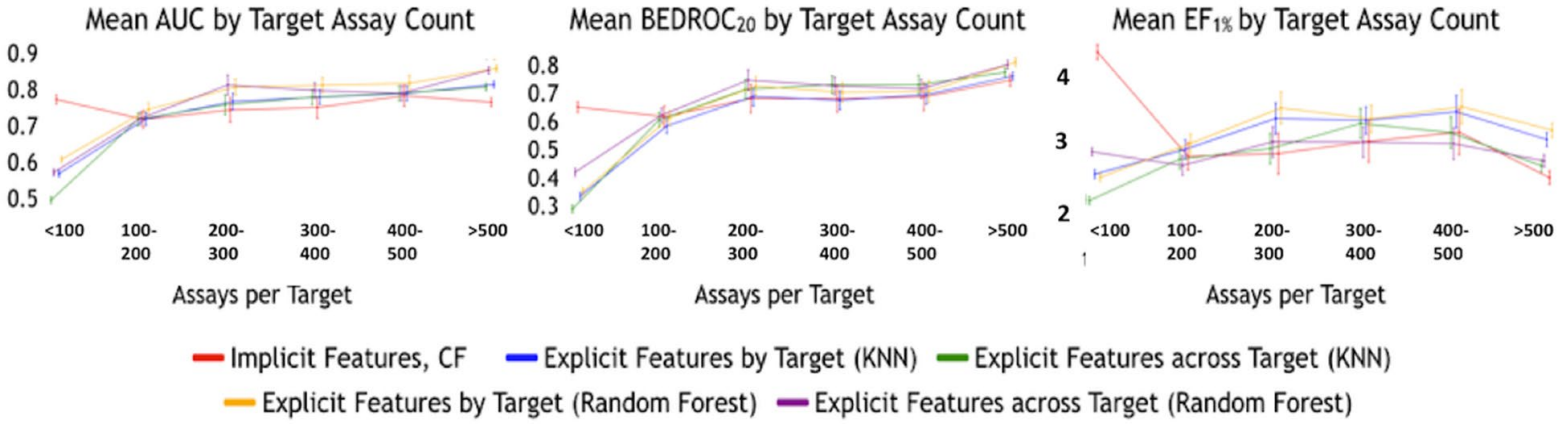
Comparison of AUC, BEDROC_20_, and EF_1%_ scores by number of known activities. Illustration of the comparative performance of the algorithms by the number of known affinities per target in the training data. Error bars correspond to standard error. The collaborative filtering based implicit structure methods significantly outperform other algorithms when the training data has 100 or fewer activities. Beyond 100 activities the performance of all algorithms converge

### Overall performance comparison

In the following section, we analyze the comparative performance of the collaborative filtering model (which uses implicit featurization) and the baseline models (which use explicit featurization from structure modeling). We use the evaluation criteria explained previously: AUC, $\hbox {BEDROC}_{20}$, and $\hbox {EF}_{1\%}$. Figure 6 shows boxplots of performance per target across all the algorithms and all evaluation criteria. That is, a performance criterion is calculated for each target and then all values are displayed in each boxplot. Results from all cross validation iterations are combined in each boxplot.

Across all evaluation criteria collaborative filtering performs similar to the baseline methods, despite the absence of known structural information of the ligands. In general, collaborative filtering is a slightly superior performer, followed by the *across target RF* model, followed by *per target RF*, and *KNN* for *across target* and *per target* rounding out the bottom. When taking the AVE Bias into account, the average AUC scores of the models generated by RF and KNN are consistent with the performance of the same algorithms on the unbiased training and validation tests for the J and J Benchmark reported by Wallach and Heifets [61].

**Fig. 8:**
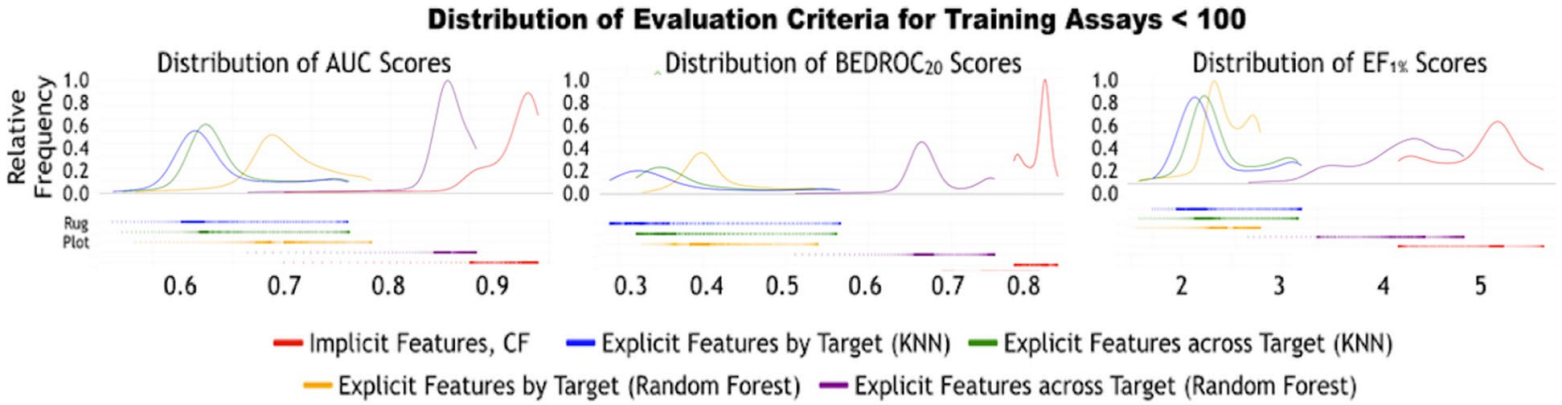
Distribution of AUC, BEDROC_20_, and EF_1%_. Distribution of AUC, BEDROC_20_, and EF_1%_ scores across all algorithms when the number of known affinities per target is less than or equal to 100 activities. Based on a two-tailed t-test of the scores ($p<0.01$), the difference in scores for collaborative filtering with other methods is statistically significant

**Fig. 9:**
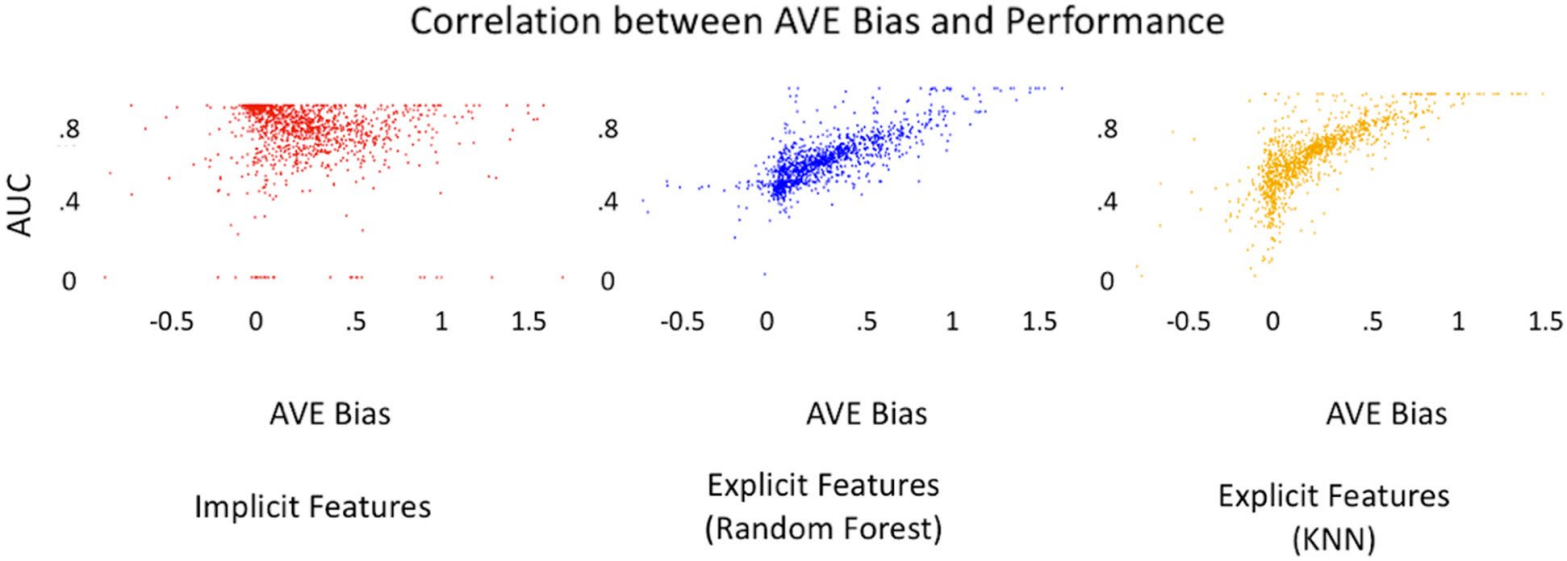
Correlation of AUC and AVE bias. Illustration of the performance of Implicit method using collaborative filtering,Random Forest and KNN algorithms using explicit features for targets with greater than 100 assays in training set. Both Random Forest and KNN show clear correlation between AVE bias and AUC while the collaborative filtering is impervious to the presence of AVE Bias.The Pearson correlation for the Random Forest method was found to be 0.69, the K-Nearest Neighbor method was 0.74, and the collaborative filtering algorithm using implicit fingerprints was 0.07

Although we have shown performance for all cross validation iterations combined, similar performance was observed individually for each validation set. Because the performance is near identical and for brevity, the “Appendix” enumerates this comparative performance per cross validation iteration.

### Performance by number of known actives

While the overall performance of collaborative filtering is encouraging, it is still unclear if the implicit features employed help to mitigate the need for large numbers of training assays for each target. To help delineate this, we now focus our attention on grouping the results by how many training assays were used to model a given target. We remind from Fig. 3 that the number of training assays per target is typically less than 200, which comprises the majority of targets in the ChEMBL database. As such, it is desirable for an algorithm to perform well even when the number of training assays is relatively low. Figure 7 shows the performance of the algorithms on the validation sets with results grouped by the number of available training assays. That is, each performance criteria is calculated for each target and, then, the results are grouped by the number of assays used in the training of that target. For example, targets with less than 100 training assays are in the first bin, 100–200 in the second bin, and so on until targets contain more than 500 training assays. We then clumped together all targets with more than 500 assays into the last bin. This approach enables us to compare the relative performance of the algorithms based on the the amount of available training assays. A consistent pattern was observed across all the three evaluation criteria. The predictions from collaborative filtering on the validation set significantly outperform the baseline methods when there were 100 or fewer assays in the training set based on a two tailed t-test ($p<0.01$). Between 100–500 training assays, the implicit feature modeling methods perform statistically no different than the baseline models ($p>0.5$). Beyond 500 training assays, the traditional baseline method using the Random Forest algorithm outperforms collaborative filtering.

It is interesting to note that the average enrichment factor in the top 1% is the highest across all methods when there are not many training assays available per target. We hypothesize that collaborative filtering becomes less effective (in terms of $\hbox {EF}_{1\%}$) when the number of ligands already tested is relatively numerous because the most likely candidates have already been assayed by chance.

**Fig. 10:**
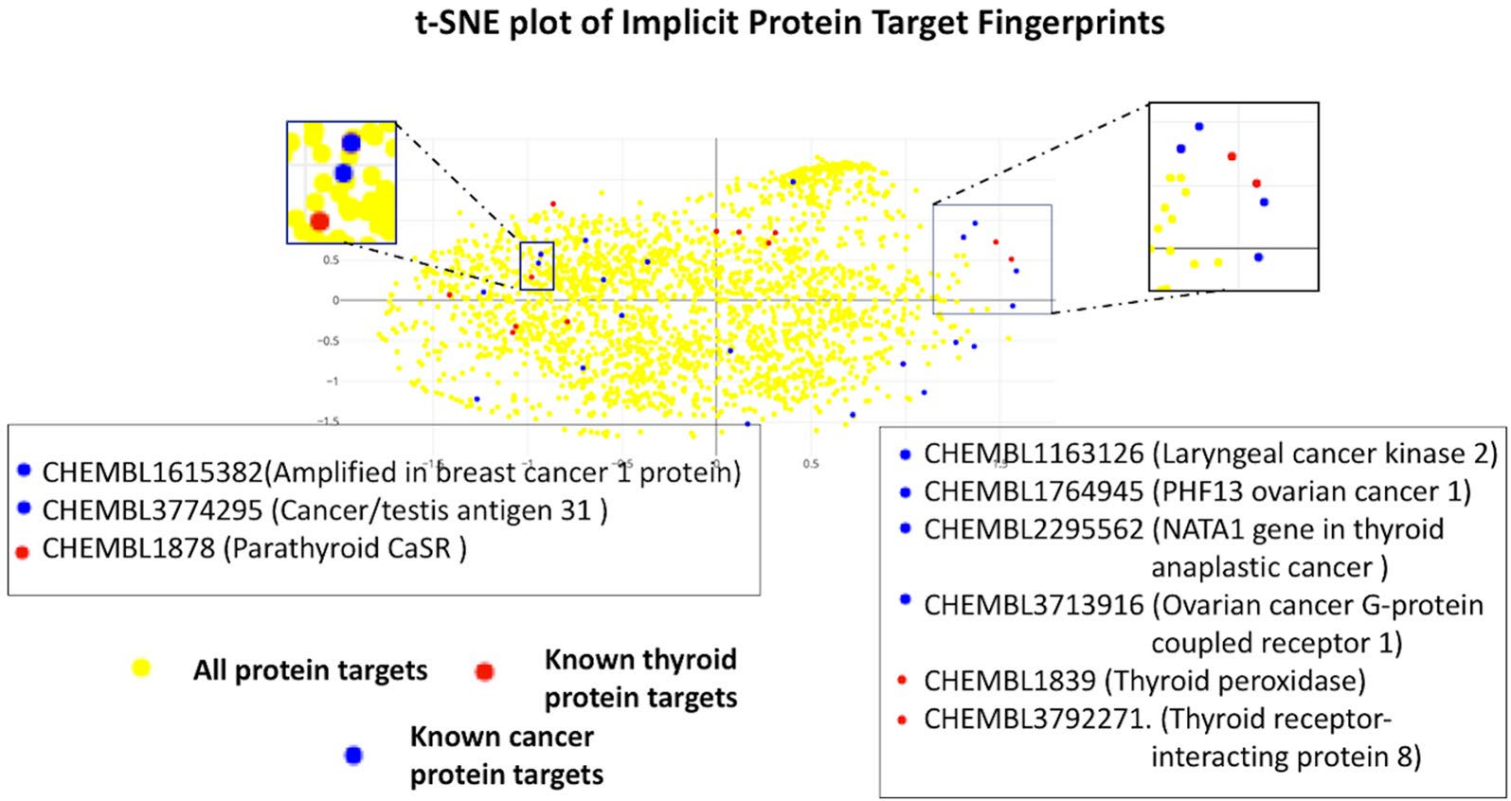
Implicit target fingerprints: t-SNE plot reducing the dimensions of the 50-dimensional implicit fingerprints into two dimensions for a subset of targets in the ChEMBL database, highlighting known cancer and thyroid related protein targets. The method successfully clusters related targets close to each other,as indicated in the zoomed version of the largest cluster of cancer targets. Interestingly the clusters also contain other targets which are found to have expressions with known cancer/thyroid targets as in the example of Vascular Endothelial Growth Factor Receptor-2 Expression in breast cancer 1 protein

**Fig. 11:**
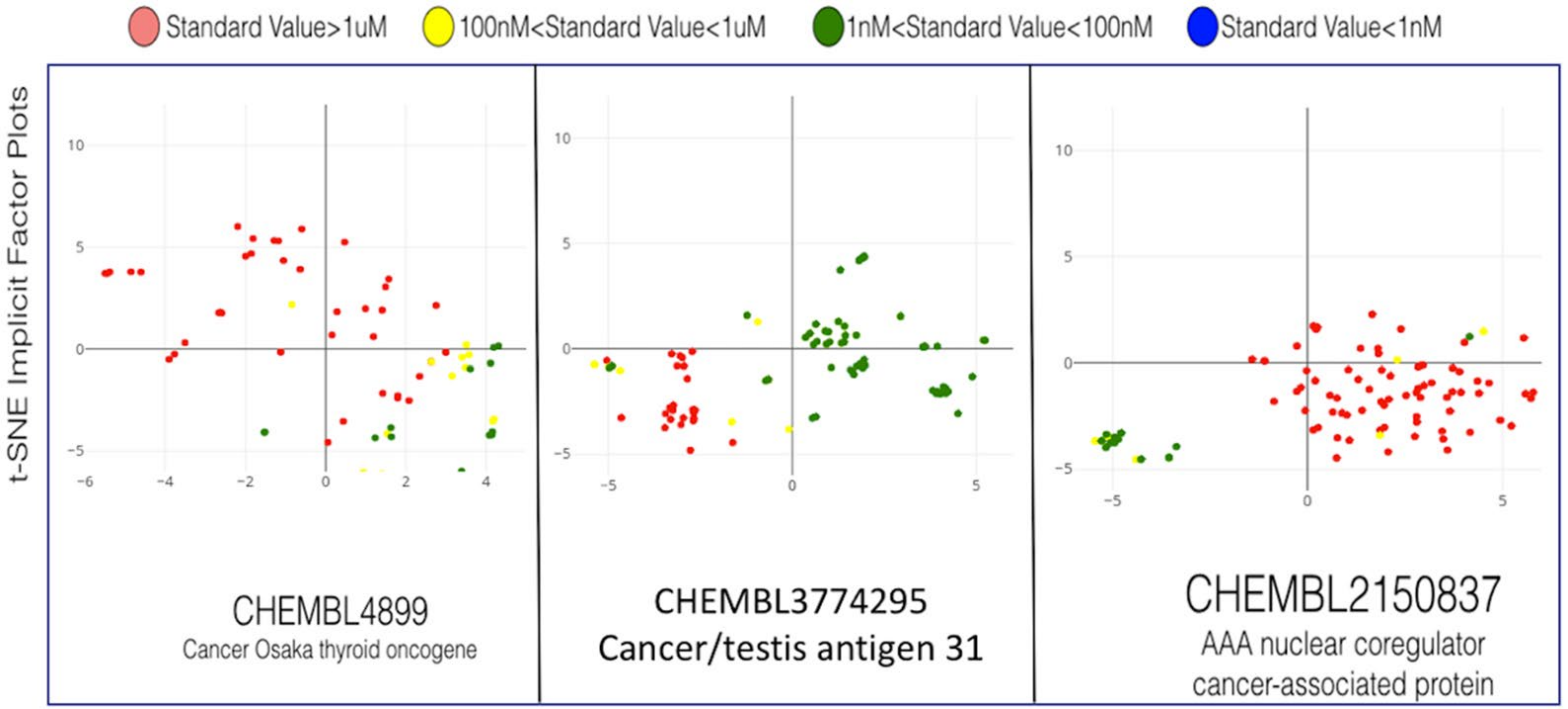
t-SNE plots of implicit ligand fingerprints. Plots for three cancer targets are shown where each point represents a compound assayed from the ChEMBL database. The concentration results of the assays are color-coded. t-SNE plots of the 50-dimensional implicit representations, reduced to 2 dimensions preserving distance

To further evaluate the differences between algorithms when the training assays are relatively sparse, we expand on the performances of the AUC, $\hbox {BEDROC}_{20}$, and $\hbox {EF}_{1\%}$ scores for targets with less than or equal to 100 training assays. Figure 8 represents the kernel density plots for the distribution of the three aforementioned evaluation criteria. Kernel density estimation is a means of estimating a smooth distribution from a finite set of points, similar in spirit to a histogram. Below each density estimate is a “rug plot” of the values for each machine learning method, with one “tick” for each observed value (this makes outliers easier to see as they can easily be hidden in kernel density estimates). The distribution of the scores from the implicit structure methods using collaborative filtering has a clear and visual separation from the other baseline methods using explicit structures. With the exception of *across target RF*, no other model has overlap in the performance for any evaluation criteria. Even so, based on a two-tailed t-test of the distributions, collaborative filtering is the significantly best performing algorithm in terms of all evaluation criteria when the number of training assays is less than 100.

We further investigated the performance of the implicit and explicit models in the context of the AVE Bias. Figure 9 illustrates correlation of the AUC scores across the implicit and explicit models with the AVE Bias for targets with more than 100 recorded assays in the training set. The Pearson correlation for the Random Forest method was found to be 0.69, the K-Nearest Neighbor method was 0.74 and the collaborative filtering algorithm using implicit fingerprints was 0.07. A lower correlation is preferable, indicating that the algorithm is robust to how training and testing sets are selected. While our results are consistent with the prior work by Wallach and Heifets [61] for the explicit methods, we conclude that the collaborative filtering methods are resilient to the AVE Bias because the predictions of the affinities are based on the interactions between the ligands and the targets rather than the molecular fingerprints. They are hence impervious to the AVE Bias in the training and the validation sets because they do not rely on “closeness” as measured by the fingerprinting process—this “closeness” is discovered through the factorization process of collaborative filtering.

Because of the clear performance separation of collaborative filtering, we conclude that the implicit structure methods demonstrate a consistent and significantly increased performance when the number of known assays is limited to about 100 assays. We also conclude that when the number of training assays is greater than about 500, traditional methods provide an increased performance. Unlike these baseline methods that use molecular fingerprinting, collaborative filtering methods can “learn” about the ligands based on their affinities with other targets and vice-versa, even with fewer numbers of known assays per target. This aspect of the collaborative filtering method contributes to the better performance even with relatively sparse assay counts.

### Implicit target and ligand fingerprints

We now turn our attention to an initial analysis of how the latent factors computed by collaborative filtering, by virtue of the information encoded within them can be used to complement the traditional molecular and target fingerprints. Therefore we introduce a new type of fingerprinting technique called *Implicit Fingerprints*,which are the latent factors determined by the collaborative filtering method on the known affinities. From our grid search, we found that 50 latent factors for each ligand and target was the most optimal performing number of factors for the matrix factorization. We further evaluate the applicability of the 50 latent factors to identify and cluster known cancer and thyroid related protein targets. Greater than 20% of industrial cancer drug development programs focus on a small subset of proteins when approximately 20,000 possible proteins are known [73]. While there have been studies investigating the pharmaceutical vulnerabilities of these proteins, the challenges with costs and off-target effects have been a limiting factor in realizing the proteins’ clinical potential. We investigated the inherent properties of the implicit fingerprints to identify targets with similar binding affinities based on prior assays results. For the purposes of the tests, we re-trained a collaborative filtering model without discarding the assays with concentration levels 100 nM and 1000 nM. The experiment was rerun as a multi-label affinity classification problem with the following labels to indicate binding affinities : $<1$ nM was associated with the affinity label of 1, between 1 and 100 nM was labeled as 2, 100 nM and 1000 nM labeled as 3 and greater than 1000 nM labeled as 4.

To visually elucidate the power of *Implicit Target Fingerprints*, we mapped the dimensions of the 50 latent factors of the targets into a 2-dimensional space using t-distributed stochastic neighbor embedding (t-SNE) [74], a powerful method for reducing dimensionality of high dimensional datasets by minimizing the Kullback-Liebler divergence of distributions in the higher and lower dimensional space. Figure 10 illustrates the distribution of the protein targets when mapped into a 2-dimensional space, with the cancer related target proteins highlighted in blue, and thyroid related in red. Interestingly, the graph demonstrates the presence of three potential clusters of known cancer related targets appearing close to each other. Similarly three potential clusters of thyroid related targets also appear close together. These cancer-related targets are visually separated from other biological targets in the latent space. The clusters also contain other targets which are found to have expressions with known cancer/thyroid targets. For example, consider the target clusters containing Vascular Endothelial Growth Factor Receptor-2 Expression (CHEMBL1878) with breast cancer 1 protein(CHEMBL1615382) or Thyroid peroxidase (CHEMBL1839) with PHD13 ovarian cancer 1(CHEMBL1764945). In the 2D implicit target fingerprints space, the close distances among these targets corresponds to known observations of similarity from prior research [75, 76]. This also engenders a potential for identifying other unexplored relations between the protein targets that appear close to each other in the implicit fingerprint space.

Similar to the biological targets, the the ligands can also be mapped in this latent space, as illustrated next. To help intuit the compound-protein binding affinity prediction capabilities of the implicit latent space, we randomly selected three cancer related targets from the ChEMBL database. We selected target with IDs CHEMBL4899, CHEMBL3774295 and CHEMBL2150837 as shown in Fig. 11. Again, the 50-dimensional implicit fingerprints of the compounds are reduced into a 2-dimensional space using the aforementioned t-SNE method. We visualize all compounds with known assays for the three selected cancer related protein targets. The compounds are color-coded on the basis of their standardized concentration levels in the assays, where a decreasing concentration level indicates stronger binding affinity. For the t-SNE plots, the ideal result would be perfect clustering for each concentration level. The implicit fingerprints from the figure demonstrate a very clear separation between the compounds based on the concentration levels required to trigger binding affinities with the respective targets. This visual separation is striking for assays with excellent binding affinity (standard value below 100 nM), indicating that the implicit representation is excellent in its ability to capture properties of similar compounds using Euclidean distance.

From the above evaluations, we conclude that the baseline modeling methods traditionally can be enhanced by the use of *Implicit Target and Ligand Fingerprints*. The models generated by the *Implicit Fingerprints* have better predictive power than their explicit counterparts when the number of known assays for training is less than 100. For the remaining targets, *Implicit Fingerprints* performs about equally to explicit molecular fingerprinting up to about 500 assays. When the number of assays is above 500, traditional methods have a slight, but significant advantage over collaborative filtering.

## Conclusions

Traditional virtual screening methods in cheminformatics have historically relied on molecular modeling of explicit ligand and protein features. However, determining all the intrinsic molecular features which contribute to bindings is a daunting, perhaps intractable task. In this work, we proposed the use of collaborative filtering to implicitly model the binding affinity between ligands and targets. We leveraged the ever-growing databases of ligand and targets binding affinity that provide a wealth of insights into various assays and their outcomes. Our study has shown that implicit structure modeling is superior to explicit structure based methods especially when the number of known assays is limited to less than 100 assays. Beyond 100 assays of training, explicit and implicit modeling approaches converge. Our study also introduced a new type of fingerprint technique generated from the latent factors found through matrix factorization in collaborative filtering. We compared the relative performance with the traditional molecular fingerprinting techniques, showing the virtual screening models trained on implicit fingerprints outperformed models with traditional molecular fingerprints especially when the training assay counts were fewer than 100 assays.

### Limitation

We conclude that implicit-descriptor modeling is a promising method for virtual screening. Even so, we point out that our analysis was completed on a large subset of the ChEMBL database, Version 23. Therefore, the consistency of implicit fingerprints for ligands across different bio-activity databases needs to be further evaluated, as well as the predictive power of the implicit fingerprints across different databases. Further studies need to be conducted on the cumulative predictive powers of the traditional ligand fingerprinting techniques and the implicit fingerprints generated from collaborative filtering in order to understand if the implicit fingerprints are consistent with other groups of targets and ligands. We also note that a limitation of our approach is that we require ligands to be assayed upon more than one target in order to evaluate them. That is, a ligand must be paired with a target in the training set and paired with another target in the testing set for our method to be able to evaluate binding affinities. We also point out that our implementation of **across target training mode** is not widely used in the cheminformatics community. We only investigated one method of fingerprinting targets using the ProFeat feature generation tool. Other target methods could provide superior performance. Moreover, we note that the Random Forest method trained using combined target and ligand fingerprinting through RDKit was typically a strong performer compared to traditional per target training (which is most often employed in the virtual screening literature). While the performance of Random Forests was inferior to collaborative filtering, this result does warrant further investigation into techniques for featurizing protein targets. Such an investigation may prove to uncover models that can perform superior to collaborative filtering methods.

## Additional files

Additional source code, results and sample fingerprint files are available at https://github.com/rsrinivas-repo/deepbind_ImplicitFingerprinting. The site contains the relevant code files for calculating AVE Bias, and training models using KNN, Random Forest and Collaborative Filtering algorithms, file contains the csv file of 50K sample ligands with their implicit fingerprints generated from the collaborative filtering algorithm and csv files with the results per target across all the evaluated algorithms.

## Figures and Tables

**Fig. 12:**
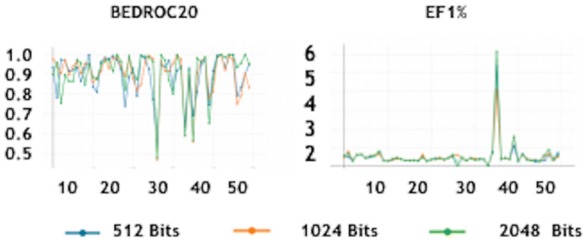
Performance of RandomForest models with molecular fingerprint Sizes 512, 1024 and 2048 BITS. Illustration of BEDROC_20_ and EF_1%_ scores on Random Forest models trained on molecular fingerprints across 3 sizes exhibit similar performance across the 3 models. We randomly chose 50 Targets and trained 3 p̈er Targetm̈odels for each Target. Each model was trained with fingerprint sizes of 512 Bits, 1024 Bits and 2048 Bits. The BEDROC_20_ and EF_1%_ scores were found to be similar across the 3 models for each Target.With little or no discernible difference in the influence of the sizes of the explicit molecular fingerprints towards the performance of the modes, we chose 512 bits to create additional models to compare performance with the collaborative filtering models for our study

**Fig. 13:**
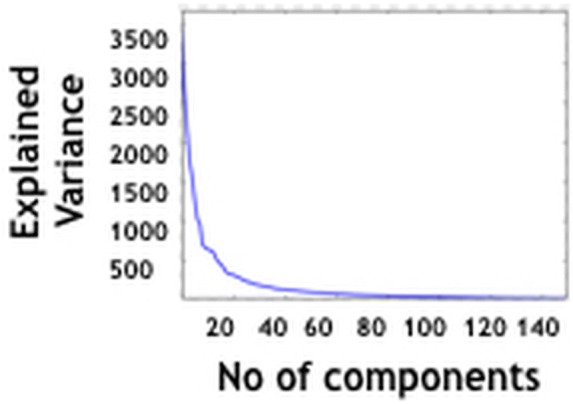
Dimensionality reduction on PROFEAT. Dimensionality reduction of 1447 Protein Features to 150 derived features

**Fig. 14:**
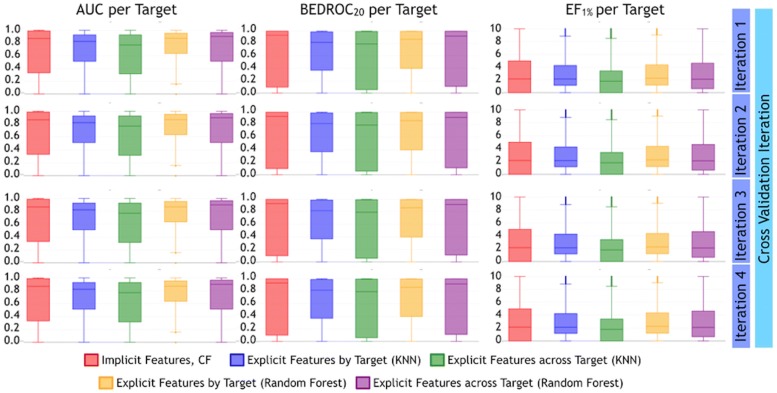
Performance across 4 iterations. Comparison of ROC, BEDROC_20_, and EF_1%_ scores across all algorithms for each iteration of the cross validation

**Fig. 15:**
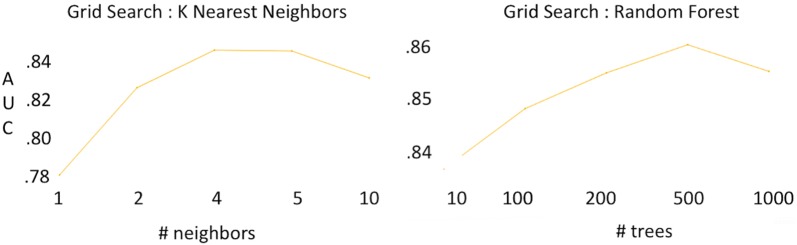
Grid search results on K nearest neighbors and random forest algorithms. Comparison of the area under the curve metric from the models trained with various hyper-parameters for K nearest neighbors and random forest algorithms

## Appendix

### Molecular fingerprinting approach

We generated RDKit ligand fingerprints of sizes 512, 1024, and 2048 bits and created per target models across 50 randomly selected targets. The purpose of this exercise was to evaluate the most optimal fingerprint size for the achieving best performance with regards to the $\hbox {BEDROC}_{20}$ and $\hbox {EF}_{1\%}$ scores. The performance of the machine learning algorithm trained on molecular fingerprints of size 512 bits, 1024 bits and 2048 bits for the 50 selected targets is shown in Fig. 12. As can be seen from the figure the performance the three machine learning models using molecule extended connectivity fingerprint [22] with the radius of two bonds (i.e., ECFP4 in RDKit package) hashed to a binary vector are similar and correlated across all evaluation criteria. Because all results were highly similar, we chose to use 512 bits for the vector size.

### Protein sequencing approach

The availability of structured representation of target proteins aids in virtual screening and serves as an alternate approach to pure ligand based virtual screening techniques. In our work, we utilize ProFeat (Protein Features) by Li et al., which is a web tool for computing commonly-used structural and physicochemical features of proteins and peptides from amino acid sequence. It computes six feature groups composed of ten features that include 51 descriptors and 1447 descriptor values. The computed features include amino acid composition, dipeptide composition, normalized Moreau-Broto autocorrelation, Moran autocorrelation, Geary autocorrelation, sequence-order-coupling number, quasi-sequence-order descriptors and the composition, transition and distribution of various structural and physicochemical properties. The protein features were generated for the 1976 protein targets which were a part of this study.

Many of the features in the 1447 features generate by ProFeat are correlated. Hence we use principal component analysis dimensionality reduction on the target features to reduce redundancy in the feature sets. Figure 13 displays the explained variance with respect to the number of derived components from principal component analysis. The explained variance plateaus around 150 derived features. As such, this study uses the 150 derived features in lieu of the 1447 features generated by ProFeat.

### Overall results across multiple iterations

The composition of the training and validation sets can influence the metrics and the performance results reported on machine learning models. It is best practice to conduct multiple iterations of training and validations to ensure consistency of the reported metrics across all iterations. We conducted four iterations of our tests. While the above sections dissect the results from the combined results of the four iterations, we present in this section the overall results from all iterations. Figure 14 exhibits the consistent performance of the algorithms across all iterations of training and validation data.

### Hyper-parameter tuning for K-nearest neighbors and random forest algorithms using grid search

We tuned the parameters for the K Nearest Neighbors algorithm and the Random Forest on the external structural fingerprints to select the best performing models using the grid search mechanism. The grid search yielded that the Random forest algorithm was most optimal when the gini index was used as the measure of impurity to split the trees. We experimented with the number of trees in the forest ranging from 10 to 1000 trees. While the performance gain was limited when moving up from a 100 to 1000 trees, we chose 500 as the number of trees in our final RF model to be consistent with previous studies [71]. For the K-Nearest Neighbor algorithm, the grid search yielded that the most optimal parameters were: using brute force distance calculations, “jaccard” distance (which is appropriate as the external fingerprints are binary vectors), and k = 5 nearest neighbors. The K Nearest neighbhors algorithm was Fig. 15 exhibits the consistent performance of the algorithms across all iterations of training and validation data.
